# Microbial membrane transport proteins and their biotechnological applications

**DOI:** 10.1007/s11274-024-03891-6

**Published:** 2024-01-16

**Authors:** Melek Özkan, Hilal Yılmaz, Pınar Ergenekon, Esra Meşe Erdoğan, Mustafa Erbakan

**Affiliations:** 1 Environmental Engineering Department, Gebze Technical University, Kocaeli, 41400 Türkiye; 2 Biosystem Engineering Department, Bozok University, Yozgat , 66900 Türkiye

**Keywords:** Aquaporins, Biomimetic materials, Formate nitrite channels, Membrane transport proteins

## Abstract

Because of the hydrophobic nature of the membrane lipid bilayer, the majority of the hydrophilic solutes require special transportation mechanisms for passing through the cell membrane. Integral membrane transport proteins (MTPs), which belong to the Major Intrinsic Protein Family, facilitate the transport of these solutes across cell membranes. MTPs including aquaporins and carrier proteins are transmembrane proteins spanning across the cell membrane. The easy handling of microorganisms enabled the discovery of a remarkable number of transport proteins specific to different substances. It has been realized that these transporters have very important roles in the survival of microorganisms, their pathogenesis, and antimicrobial resistance. Astonishing features related to the solute specificity of these proteins have led to the acceleration of the research on the discovery of their properties and the development of innovative products in which these unique properties are used or imitated. Studies on microbial MTPs range from the discovery and characterization of a novel transporter protein to the mining and screening of them in a large transporter library for particular functions, from simulations and modeling of specific transporters to the preparation of biomimetic synthetic materials for different purposes such as biosensors or filtration membranes. This review presents recent discoveries on microbial membrane transport proteins and focuses especially on formate nitrite transport proteins and aquaporins, and advances in their biotechnological applications.

## Introduction

**Fig. 1:**
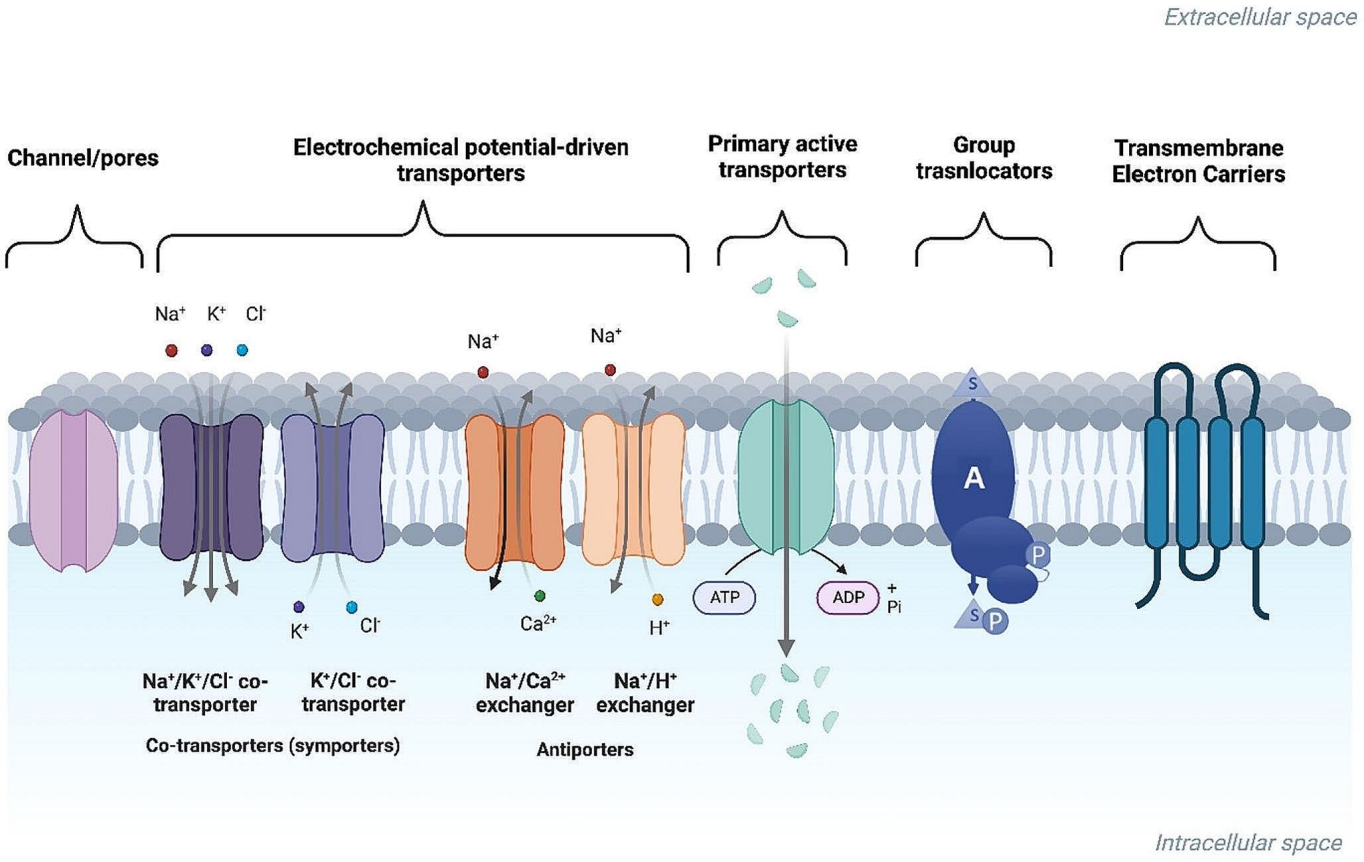
Types of membrane transport proteins

The cell membrane separates the interior part of cells from the outside environment. It consists of a phospholipid bilayer with embedded proteins (Mishra et al. 2016). Transport of molecules through the membrane occurs via simple diffusion, facilitated diffusion, active transport, and endocytosis (Paul 2019; Wu et al. 2023). Most molecules have to be transported across the cell membrane with the help of intrinsic or extrinsic transmembrane proteins. There is an enormous number of channels, pumps and carriers found in different organisms, many of which are comprehensively discussed by Stein and Litman (2014). Detailed categorization and knowledge about different types of microbial transport systems was performed by Winkelmann (2001). Different categorizations of membrane transport proteins (MTPs) exist in the literature. The most comprehensive classification of transport systems can be found in The Transporter Classification Database (TCDB). It subdivides MTPs into nine main superfamilies which includes channels/pores, electrochemical potential-driven transporters, primary active transporters, group translocators and transmembrane electron carriers (Saier et al. 2021) (Fig. 1). There are also accessory factors in transport and incompletely characterized transport systems in the list. Channels and pores family involves different channel-type facilitators including α-type channels, which are ubiquitously found in the membranes of all type of organisms, and β-barrel porins with their β-strands spanning the breadth of the outer membrane of Gram-negative bacteria (Nikaido 1992). In order for many molecules to effectively cross the outer membrane, they must enter through these porins. Maintaining osmolarity, salt, and nutrient transport and contributing virulence are other functions of the porins (Donev 2022). The study investigating structures of beta-barrel porins including *Escherichia coli* LamB, OmpA, OmpC, and OmpF shows that these porins have mosaic evolution patterns resulting in high variability in their external parts. Interestingly, these regions coincide with the binding sites of bacteriophages (Chen et al. 2022) which emphasizes another function of the porins like rapid avoidance of the invasion of phages or antibiotics.

Active transporters which are also known as TonB-dependent transporters, and channel type facilitators like channels and porins modulate flux through the membrane by selecting the molecules according to their size, chemical composition, and charge. Unlike porins and channel proteins, these membrane transporters change their conformation while transporting the solute to the other side of the membrane (Alberts et al. 2002). They can also couple uphill substrate translocation with the movement of ions down their electrochemical gradient, or by ATP hydrolysis. These processes enable bacteria to scavenge nutrients that may be scarce (Nikaido et al. 1992; Davies et al. 2021). Transporters with low or high specificity also function in passive diffusion of metabolites helping facilitate diffusion through the membrane. Low-specificity transporters can also be called mechanosensitive channels since they open mechanically due to the swelling of cells in the hypoosmotic environment (Wang et al. 2014). *Corynebacterium glutamicum* MscCG, responsible for glutamate excretion is an example of that kind of facilitated transporter. The lysine uptake system of the same bacterium is on the other hand a kind of active transporter that is powered by the externalization of other amino acids (Broer and Kramer 1990; Becker et al. 2013).

In the present review, channels, porins, and transporters especially important for biotechnological applications are discussed mainly. The idea of the existence of molecular water channels that permit the osmotic flow of water across membranes belongs to Koefoed-Johnsen et al. (1953). The earliest studies on membrane transporters are on porins of Salmonella and *E. coli* (Nakae 1976; Nikaido and Rosenberg 1981). The other examples of early studies on transport proteins are on MalKGFE maltose transporter (Szmelcman et al. 1976), which is a type of primary active transporter depending on ATP hydrolysis and LacY lactose permease, a secondary transporter needing an electrochemical gradient (West and Stein 1973). The transporters which do not need energy usually transport water or ions. *E. coli* GlpF glycerol channel (Sweet et al. 1990), aquaporins of many different bacteria such as *E. coli* Aqp Z(Calamita et al. 1995) and *Halomonas elongata*(Çalıcıoğlu et al. 2018) and nitrite/formate transporters from various bacteria including *S. typhumirium* and *E. coli*(Rycovska et al. 2012; Yılmaz et al. 2023) can be given as examples to these facilitated transport proteins. The nitrite transporter (NirC), the formate efflux transporter (FocA), and other members of the formate-nitrite transporter (FNT) family found in bacteria, archaea, and yeasts are categorized under α- type channels.

Apart from the membrane transport proteins already discussed, a class of auxiliary proteins known as viroporins might be considered prospective targets for pharmaceuticals. Viroporins are a varied class of multifunctional proteins that are found in a wide range of viral families, with a focus on RNA viruses (Nieva et al. 2012). They are usually made of 50–120 amino acids and commonly take on tetrameric structures when they form homo-oligomers (Wang et al. 2011a). These hydrophilic porins are selectivity filter that allows ions or tiny solutes to flow through the membranes of the host cell along their electrochemical gradient. They also have specified charge selectivity and translocation efficiency (Breitinger et al. 2022). Viroporins actively contribute to several of the functions of viruses, most notably the promotion of viral particle release from cells. Additionally, the glycoprotein transport system, membrane permeability, and cell vesicle systems are all impacted by these proteins (Nieto-Torres et al. 2015). Viroporins are not required for virus replication, although they do typically promote viral proliferation when they are present. In order to improve contact with the interfacial lipid bilayer, certain viroporins may additionally include other motifs, such as domains rich in aromatic amino acids or basic amino acid residues. Hydrophilic holes appear in the membranes of virus-infected cells as a result of viroporin oligomerization (Farag et al. 2020). Influenza A virus (IAV) M2, the first and most studied viroporin, was discovered in 1992 (Pinto et al. 1992). Later, other viral ion channel proteins were found in additional dangerous animal viruses, including the coronaviruses (CoV), HIV-1, and hepatitis C virus (HCV) (Sze and Tan 2015).

### Structure and transport mechanisms of MTPs

Unlike water-soluble proteins, it is hard to study membrane proteins in vitro since they expose their hydrophobic residues to the membrane, instead of burying them in the protein interior. Their hydrophilic residues are found outside the membrane interacting with different hydrophilic residues or neighboring lipid headgroups at the membrane edges, sometimes inside of the protein structure, like in the case of channels (Harris and Booth 2012). The structure and positioning of different membrane transport proteins are illustrated in Fig. 1. Transporters often have more than one domain or even multiple subunits, which create further difficulties in vitro studies (Lemieux 2008; Boudker and Verdon 2010; Harris and Booth 2012). For the investigation of their structures, crystallization and X-ray crystallography are applied. Commonly, engineered proteins fused to tags for purification, which are frequently truncated at their termini, are used for transport mechanism studies (Bill and Hedfalk 2021). Although these studies are very helpful for enlightening the solute specificity and transport mechanisms of MTPs, the problems are still encountered during their heterologous expression and purification such as low levels of expression and the need for some interfering detergents for their extraction from the membrane. The necessity of their re-alignment in liposome structures due to the lack of activity outside of the membrane is another challenging side of studying MTPs. Stopped flow light scattering spectroscopy is commonly used for studying substrate specificity of the channels and pores placed in liposome structures (Yılmaz et al. 2023; Kumar et al. 2007; Borgnia et al. 1999). Lipid bilayer electrophysiology (Lü et al. 2012) and cryo-electron tomography (Chang et al. 2023) are also used for the functional and structural characterization of bacterial MTPs. Cryo-electron microscopy has been proven to be a powerful tool for studying the structures of intricate membrane proteins that were previously intractable using other methods like X-ray crystallography. The recent improvements in cryo-EM technology made it possible to investigate the molecular mechanism of many previously intractable integral membrane proteins at the atomic resolution level (Nygaard et al. 2020).

The transport mechanisms and solute specificity of MTPs could be revealed by using the above-mentioned methods. Although it is regarded that membrane transporters are specific to their solutes and they mostly transport a specific ion, sometimes two, their selectivity is not perfect (Ansoborlo and Adam-Guillermin 2012). For example, there is a significant sequence homology in bacterial members of the formate nitrite transporter (FNT) family, and they probably transport structurally related oligoatomic anions, such as formate and nitrite (Rycovska et al. 2012).

Many studies are reporting the low specificity of porins allowing passage of diverse small hydrophilic molecules (Mckinlay 2023). For some of the nutrients, bacteria may not even need these porins for basal growth. It was recently discovered that a *Pseudomonas aeruginosa* mutant lacking all 40 porins was able to grow on some nutrients like the wild type did (Ude et al. 2021). *Mycobacterium tuberculosis* which has one of the most rigid cell envelopes lacks the classical porins. When a fast-growing non-pathogenic mycobacteria’s heterologous MspA porin was expressed in *M. tuberculosis*, its virulence traits decreased and its susceptibility to antibiotics increased considerably (Mailaender et al. 2004; Lamrabet et al. 2014). Recent research indicates that these slow-growing mycobacteria may have substituted some PE/PPE family proteins for porins as molecular transport channels to enable the uptake of nutrients necessary to live in the constrained host environment. Although bacteria may change their transport mechanisms and find a way to cope with the lack of porins their balance in cell machinery depends on the proper functioning of the transport system. The lack of different porins in *E. coli* significantly elevated the amounts of fatty acids and phospholipids and also caused structural changes in protein and DNA (Kilicaslan et al. 2023). Also, secondary structures of these channels are very important for their function and three-dimensional architecture and localization in the membrane. For example, lysine residue changed with arginine results in lower conductance in OprP and OprO porins (functional in selective uptake of phosphate molecules) of *P. aeroginosa* (Piselli et al. 2023).

There are an enormous number of microbial membrane transporters which have unique structural properties. In this review, details of the structure of transport proteins and the transport mechanisms have been tried to be presented by focusing on two different channel proteins, aquaporins and FNTs.

### Aquaporins

**Fig. 2:**
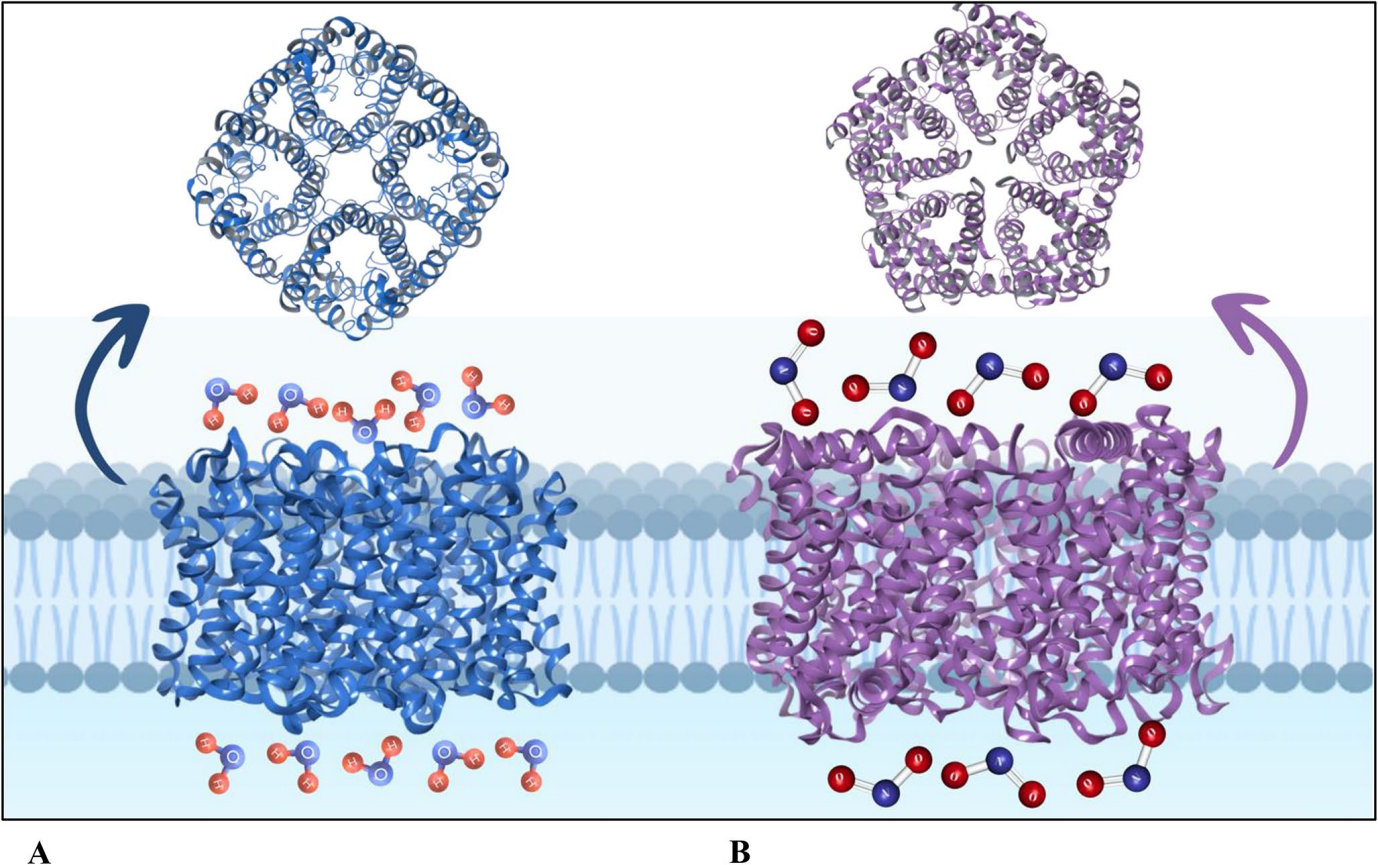
Structure and membrane localization of (**A**) aquaporin and (**B**) nitrite channel protein (NirC, a member of the FNT family) of *E. coli*. Although monomers of FNT channels and aquaporins are similar in their positioning in the cell membrane and the topology of the six transmembrane helices is conserved, small alterations in the monomer cause a significant change in the quaternary structure: FNT channels remain stable as pentamers, but aquaporins behave as tetramers (Lü et al. 2012)

Aquaporins are integral membrane proteins facilitating the transport of water and sometimes other small molecules across the lipid bilayer (Tong et al. 2019). They fold into an hourglass shape forming tetramers in which each subunit forms a central pore allowing the water molecules through while rejecting the protons, hence the name aquaporin (Fig. 2A). They have six transmembrane α-helices and two NPA (asparagine-proline-alanine) motifs responsible for the formation of an hourglass shape and Arg residue by the narrowest point of the selectivity filter rejecting the charged solutes. They typically form tetramers, while oligomerization is not essential for water transport activity (Verkman 2013). Aquaporins have a highly efficient selectivity filter achieving both size exclusion and charge repulsion. They are abundant among all living organisms from prokaryotes to mammals and responsible for fast water transport rates of red blood cells and renal tubules. Along with their conventional roles in water transport, aquaporins have been linked with the transport of other small solutes including glycerol, H_2_O_2_, urea, O_2,_ and CO_2_. Several reports describe aquaporins with lactate permeability (Schmidt et al. 2021).

The discovery of human aquaporins pioneered the discovery of aquaporin orthologs in other species ranging from microbes to plants (Azarafza et al. 2023). Microbial aquaporin genes have been identified in mollicutes, Gram-positive and negative bacteria, Archea, yeast, and mold. The first identified microbial aquaporin was AqpZ from *Escherichia coli*, which was held accountable for the adaptation of the bacterium in hypoosmotic conditions and rapid growth in the logarithmic growth phase (Calamita et al. 1998). Subsequent reports emphasize the roles of microbial aquaporins are rather specific to the growth environment of species such as tolerance for rapid freezing (Tanghe et al. 2004) and CO_2_ transport (Ding et al. 2013).

In addition to controlling water transport across plasma membranes, aquaporins have been associated with other important vital functions such as cell migration, diarrhea, and cancer cell proliferation (Ishibashi et al. 2011). They are targeted by certain drug families and employed as reporter genes for monitoring the recombinant protein expression via diffusion-weighed MRI.

Although structures of FNTs and aquaporins have very similar protein folds (Fig. 2), FNTs facilitate weak acid anion/H^+^ cotransport, whereas AQP water channels strictly exclude charged substrates including protons. Schmidt and Beitz (2022) analyzed the protonation status of the central histidine during substrate transport by mutation of this residue. Constrictions-widening mutations revealed that enlargement of the constrictions in FocA of *E. coli* exhibited water permeability similar to AQPs.

### Formate/nitrite transporters (FNTs)

FNTs are a family of membrane intrinsic proteins that allow the passage of monovalent anions. Like the other known AQP and monocarboxylate transporters (MCT), FNT also makes material transport possible via electrostatic attraction. While AQPs are ubiquitous, FNT exists in only microorganisms including prokaryotes (bacteria and archaea) and lower eucaryotes. This property actually gives rise to its use in drug targets for pathogens in the human body or more generally in mammals who do not have FNT in their cell membranes.

The most known FNT subfamily types are FocA for formate (Suppmann and Sawers 1994), NirC for nitrite (Clegg et al. 2002), HSC for hydrosulphide, and PfFNT for lactate transport (Wang et al. 2009; Lü et al. 2012). Although they are named after the main anion, they are capable of translocation of other small anions and weak organic acids. There are also two clusters under the YfdC subfamily YfdC-a and Yfdc-b, however, they are uncharacterized, and their functions are not identified yet. A recent computational study by Mukherjee et al. (2020) suggested that YfdC-a might be responsible for the translocation of neutral or cationic substrates (Mukherjee et al. 2020).

FocA of *Escherichia coli* is the first FNT channel protein to be identified (Suppmann and Sawers 1994). Saier et al.’s phylogenetic characterization revealed that NirC from *E. coli* and *Salmonella typhrium* is a possible nitrite transporter, FdhC from *Methanobacterium formicicum* a probable formate transporter (Saier et al. 1999). Saier’s database for FNTs sharing the FNT models and basic details of them had only a couple of sequenced proteins of the FNT family in the ‘90s, however, today there are more than 4000 sequences for FocA alone.

In the coming years, NirC as a nitrite channel was identified by Clegg et al. (2002). NirC functions as an importer of nitrite anions. It translocates nitrite from the periplasm to the cytoplasm, where nitrite is reduced to ammonium by the nitrite reductase NirBD. NirC is encoded in the same operon as NirBD, and their physiological roles are mainly in nitrogen assimilation and detoxification.

HSC (also named FNT3 or AsrD) is very similar to NirC and part of the assimilatory sulfite reduction pathway, where it removes the toxic end product hydrosulphide from the cytoplasm. While in the literature, it can be seen much research concentrated on the NirC and FocA subfamilies from different bacteria, on HSC there is only one study in which Czyzewski and Wang (2012) characterized HSC from *Clostridium difficile.*

The top view of FNT channels which belong to FocA of *S. typhimurium* shows that they form stable pentamers in the cytoplasmic membrane (Lü et al. 2011). The channel structure contains five individual pores in which the translocation takes place (Fig. 2B). In the FNT structure, there are two narrow constrictions on both ends of the central vestibule, one on the periplasmic side and the other on the cytoplasmic side. FNT channels overcome the hydrophobic barrier to transport anions through a mechanism that involves a histidine residue. This histidine residue plays a key role in the transport process by transiently protonating the transported anion.

FNT proteins anion translocation mechanisms have been studied largely for different family members(Suppmann and Sawers 1994; Rycovska et al. 2012; Lü et al. 2012; Beyer et al. 2013; Hunger et al. 2014; Erler et al. 2018) and FocA and other FNTs are classified as channels but there were studies in the literature reporting FNTs also as a transporter. For example, a family member of FocA was first shown to take the role of only exporting the formate ion from the cell cytoplasm, while NirC and HSC, can provide bidirectional transport (Lü et al. 2012). However, later **t**he structures of FocA obtained from different bacteria showed that they are very similar in TMS packing to AqpZ and Glycerol channel GIpF.

The studies on the mechanism of the transport of anions are very crucial in terms of both providing an understanding of the role of FNTs in changing conditions and developing biotechnological products aiming at specific membrane gate automation. Schmidt and Beitz (2022) for example tried different mutations on areas controlling the constriction-widening which is vital for the transport process.

Identifying new FNTs and understanding the details of the mechanisms of known FNTs have high potential in improving the efficiency of bioproduction processes in the biotechnological industry, developing effective drug targets as well as designing highly selective separation and efficient purification systems.

### Biotechnological applications of membrane transport proteins

**Fig. 3:**
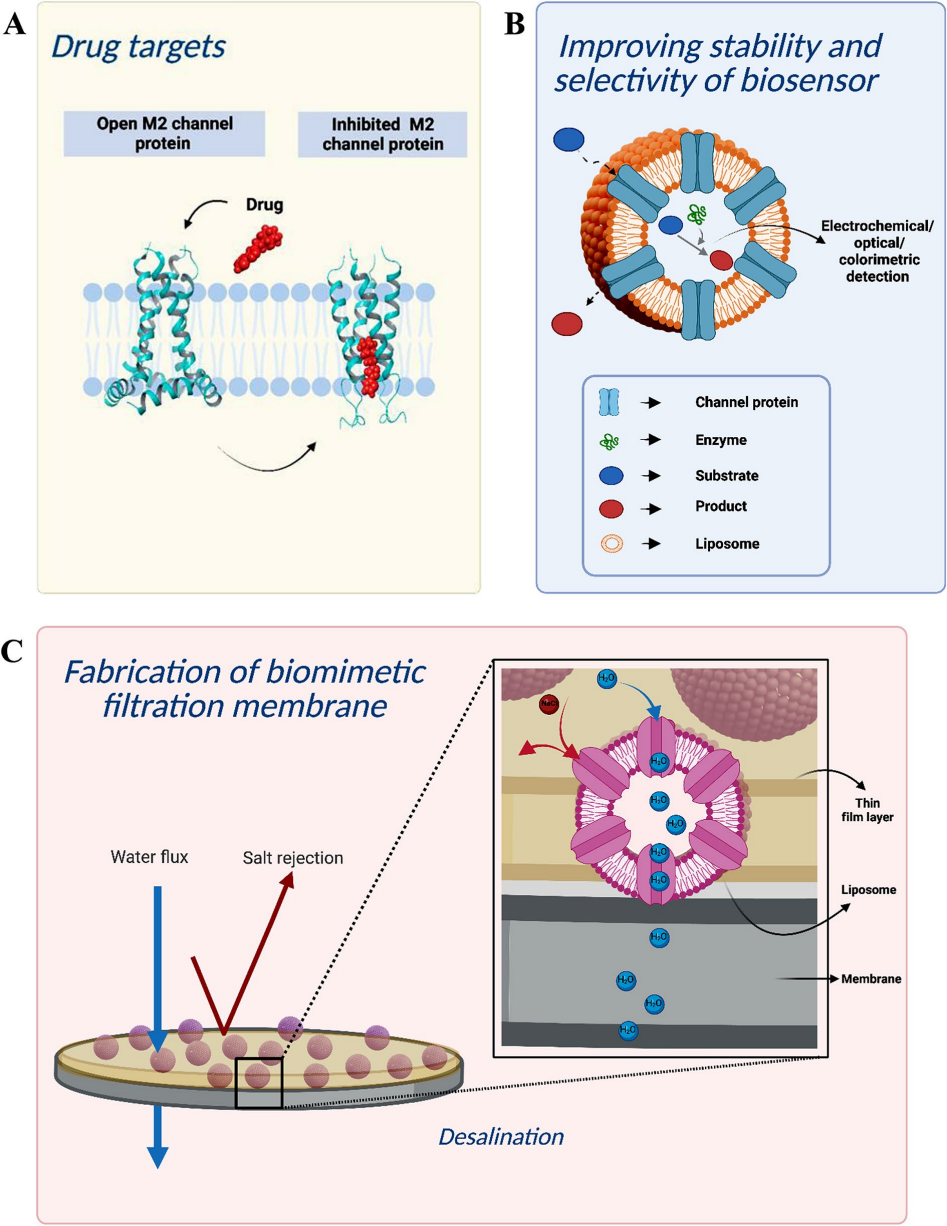
Biotechnological application examples for microbial transport proteins (**a**) novel drugs targeting MTPs; (**b**) use of channel proteins for entry of substrate in liposome-based biosensors (**c**) usage of aquaporins for increasing water flux through filtration membranes

Interest and research on the structure and function of channel proteins are increasing each day since their potential for biotechnological applications and the production of value-added substances has been noticed. In this part, potential application areas are given and the importance of different transporters for industrial applications is discussed. The fabrication of biomimetic filtration membranes, the development of biosensors using the advantages of porins, and the targeting of MTPs for disease treatment are several of these studies (Fig. 3). Table 1 lists the examples of MTPs and their biotechnological application areas. Most of the research in this table are lab scale experiments and several of them are presented as possible future applications. Unique properties of MTPs also lead the design and production of nature-inspired transport control systems.

**Table 1:** Examples of MTPs and their use for biotechnological applications

Protein type	Transmembrane protein/PDB ID	Source microorganism	Selectivity	Biotechnological application area	Referance
Outer membrane protein (porin)	LamB 1MPN	*Vibrio sp*	nonspecific porins	vaccine candidate among the vibriosis	(Lun et al. 2014)
	OmpF 3K1B	*Escherichia coli*	nonspecific porins	liposome-based nano-biosensor	(Yan et al. 2013)
	CarO 4FUV	*Acinetobacter baumannii*	carbapenem associated outer membrane protein	drugs targeting	(Gopikrishnan and George Priya Doss 2023)
	TbuX 3BRY	*Ralstonia pickettii* PK01	toluene selective	monoaromatic hydrocarbon degradation	(Hearn et al. 2008)
	AltL 3DWO	*Acinetobacter venetianus* Rag-1	alkane transporter	crude oil degradation	(Liu et al. 2022a)
	FadL 1T16	*Vibrio cholerae*	long-chain fatty acids	long-chain fatty acid transport, petroleum degradation	(Turgeson 2022)
	MspA 1UUN	*Mycobacterium smegmatis*	beta barrel porin	machine learning assisted structural profiling of proteins	(Liu et al. 2022b)
				mapping engineering sites of channel for nanoreactor configuration	(Zhang et al. 2021)
Channel-type transporters	Cch1p/Mid1p -	*Saccharomyces cerevisiae*	Ca^2+^ion channel	improving ethanol production	(Dong et al. 2021)
	AqpZ 2ABM	*Halomonas elongata* *Escherichia coli*	water channel	biomimetic membrane (micropollutant removal, desalination)	(Yılmaz and Özkan 2022; Çalıcıoğlu et al. 2018; Zhao et al. 2012)
	α-Hemolysin 3ANZ	*Staphylococcus aureus*	mitochondrial voltage-dependent anion channel	length determination of DNA and RNA	(Kasianowicz et al. 1996)
	MscCG 6PWP	*Corynebacterium glutamicum*		L-Glutamate production	(Wang et al. 2018; Wen and Bao 2019)
Carrier proteins	NirC (channel) 4FC4	*Salmonella typhumirium*	uni-, sym-, and antiporters	drug target for pathogenic *Salmonella* strains	(Wiechert et al. 2017)
	PfFNT 6VQQ	*Plasmodium*	lactate transporter	targeting lactate transporter of PfFNT of the parasite, strategy to combat malaria.	(Wu et al. 2015) (Golldack et al. 2017; Walloch et al. 2021; Davies et al. 2023)
	TonB 2GRX	*Escherichia coli*	iron ion channel	microbial biosensors to detect specific metals	(Cuero et al. 2012)
Viroporin	Gramicidin A 1MAG	*Brevibacillus brevis*	antibiotic peptide	rapid detection of influenza A virus	(Oh et al. 2008)
	M2 2L0J	Influenza A	membrane-spanning tetrameric proton channel	antiviral drugs amantadine and rimantadine	(Cady et al. 2010)
	P7 2M6X	Hepatitis C	proton channel	target for drugs (hexamethylene amiloride)	(Premkumar et al. 2004)
	Vpu 1PI7	HIV-1	proton channel	target for BIT225	(Khoury et al. 2010)
	E channel Orf3a viroporin 6XDC	SARS-CoV-2	transport viral component within the infected host cell	flumatinib treatment reduces SARS-CoV-2 RNA levels	(Singh and Arkin 2022)
Artificial channels	peptide-appended hybrid [4] arene (PAH [4]) -	*-*	artificial water channels (AWCs)	alternative to natural water channels	(Song et al. 2019)
	T-channel -	*-*	mimicking the natural Gramicidin-A	proton/water conduction, cation/anion selectivity and large open channel-conductance	(Barboiu et al. 2014)

### MTPs as drug targets and tools for gene therapy

One of the most important application areas of membrane transport proteins is exploring novel drugs for the treatment of infectious diseases. Most of the antibiotics target cell wall or protein synthesis and they are widely used for the treatment of diseases caused by pathogenic bacteria. However, novel antibiotics must be discovered, or modified forms of common antibiotics have to be synthesized due to the development of resistance against them. MTPs of pathogens have important roles in resistance development against antibiotics since most of the antibiotics have to interact with porins and pass through the channel to exert their activity (Prajapati et al. 2021). For example, OprD is a substrate-specific porin that facilitates the diffusion of basic amino acids, small peptides, and carbapenems (Trias and Nikaido 1990). Decreased expression of this porin was shown to be related to resistance to antibiotics in some pathogens including *Acinetobacter baumannii* and *Pseudomonas aeroginosa* (Strateva and Yordanov 2009; Ebrahimi et al. 2023). OprD of these bacteria which have mutations in their DNA sequence had a unique porin diameter and decreased anion selectivity compared to the wild-type strain (Ebrahimi et al. 2023). Efflux pumps are also found to be related to the multidrug resistance mechanisms of *A. baumannii* (Sharma et al. 2023). Another well-known porin in *A. baumannii* is CarO (carbapenem-associated outer membrane protein) which was reported to contribute to the resistance to carbapenem antibiotics. Potent drug candidates with a better binding affinity to CarO have been identified with molecular docking and dynamic simulation study (Gopikrishnan and George Priya Doss 2023). Understanding the ion mobility across the channels can potentially lead to the development of more effective drug molecules (Piselli et al. 2023). Interestingly, these outer membrane proteins are induced in high numbers if the bacteria are in contact with lung epithelial cells (Chugani and Greenberg 2007; Chevalier et al. 2017), which results in higher resistance to common antibiotics.

Novel drugs targeting membrane transport proteins are being discovered and they have high potential to be used for disease treatment. FNT channel proteins can be utilized as a drug target since human cells do not have these special channel proteins in their membrane making them perfect targets for killing the parasites causing diseases in humans. In 2015, the *Plasmodium* lactate transporter of PfFNT was identified almost concurrently by two studies (Wu et al. 2015; Marchetti et al. 2015). PfFNT was shown to be responsible for the lactate transport in *Plasmodium.* Glycolytic oxidation is critical for rapidly proliferating cells to create effective virulence. Glycolysis therefore requires very fast glucose influx and two times faster lactate efflux. Targeting lactate transporter of PfFNT of the parasite would be then a viable strategy to combat malaria. The study proved that PfFNT of *Plasmodium* takes lactate in ion form and co-transports it together with a proton (symport of Lactate/Proton) and this bi-directional transport can be inhibited by diethylpyrocarbonate (DEPC) (Wu et al. 2015). Therefore, being in only microbial cell membranes, PfFNT was identified as a putative drug target for malaria. Later it was shown that PfFNT indeed is a valid drug target in follow-up studies by many researchers (Golldack et al. 2017; Walloch et al. 2021; Davies et al. 2023).

Another parasite, *Toxoplasma gondii’*s FNT proteins in the plasma membrane were also studied and three FNTs (TgFNT1, TfFNT2, and TgFNT3) are identified which can transport both lactate and formate bidirectionally (Erler et al. 2018). The use of 2-hydroxy chromanones is shown to inhibit the transport of these ions and is suggested as putative drug targets. However, Zeng et al. (2021) showed that these TgFNTs are not critical in the parasite’s rapid growth and therefore they may not have strong potential to be used as a drug target for Toxoplasma (Zeng et al. 2021). Helmstetter et al. (2019) identified EhFNT as the sole FNT in *Entamoeba histolytica*, a parasite that causes intestinal illness known as amoebiasis. The identified EhFNT can be further studied to explore its potential to be used as a new drug target (Helmstetter et al. 2019).

Designing or selecting antimicrobial agents targeting specific MTPs of unwanted biofilm-forming bacteria may be another strategy for disease treatment. In a recent study, it was revealed that biofilm formation is related to the nitrite transporter since it regulates the NO amount which can cause both biofilm dispersal and formation. This property therefore can be used in producing antibiofilm agents preventing the formation of biofilm which helps the pathogens’ antibiotic resistance (Park et al. 2020). Thus, this study identifies nitrite transporters as new antibiofilm targets for future practical and therapeutic agent development.

Bacterial MTPs bring advantages over mammalian MTPs because of their small size and easy manipulation. Also, drugs targeting mammalian MTPs have the risk of harming the eukaryotic cells of the host. For example, gene-based therapies involving voltage-gated sodium channels are largely hampered by the large size of the mammalian channels. Fortunately, voltage-gated ion channels (BacNavC) harboring many of the core features of eukaryotic ones (responsible for high ion flux) are also discovered in bacteria (Payandeh and Minor 2015). They are regarded as a target for pharmaceutical drugs (McCusker et al. 2012). It was found that prokaryotic sodium channels (BacNav) cloned under muscle-specific promoters significantly enhanced excitability and conduction in rat and human cardiomyocytes in vitro (Nguyen et al. 2022). Another example of a possible gene therapy application is the use of rhodopsins for the treatment of neurological disorders (Ji et al. 2013). Because of their ability to interact with light, rhodopsin and rhodopsin-like ion channels have been extensively studied and they are ideal targets for fluorescence-based investigation (Islam et al. 2021). Among these ion channels, channelrhodopsins and halorhodopsins have been used in optogenetic applications, such as modulating neuronal activity and blocking different cell types in the intact nervous system (Gradinaru et al. 2008).

### Improving metabolic activities and p**r**oducing industrially important p**roducts**

It is reported that membrane transport proteins are undervalued, and their manipulation actually can provide specific control on the flux through the cell in the name of increasing the efficiency of bioproduction (Kell et al. 2015). Although these transporters are seen as potential targets for medical and biotechnological applications, the high number of the reported membrane transporter families in the literature are poorly characterized (Radi et al. 2022). One of them is the membrane transport systems involved in the degradation process of petroleum hydrocarbons (Wu et al. 2023; Hua and Wang 2014). It is reported that the transport of these molecules is regulated by various gene families such as the FadL family, the OmpW family, ABC-type transporters, and TonB-dependent transporters (Wu et al. 2023). Many export pump systems for aromatic hydrocarbons have also been studied, especially for *Pseudomonas putita*, *E coli*, and *Pseudomonas fluorescens* (Mutanda et al. 2022) (Table 1). The discovery of novel transport proteins for pollutants and exploration of their characteristics will accelerate the research on the production of efficient recombinant microorganisms and allow more effective bioremediation applications in the future.

The biological production path offers a cost-effective and more sustainable production compared to the common method of petroleum-based chemical production. Identification of the roles of specific membrane transporters has opened up a vast area of opportunities in engineering these gates for specific conditions or substrates such as organic acids (Van Dyk 2008; Kell et al. 2015). There is a quite high number of research on the genetic modification of transport proteins to obtain industrially valuable microbial strains. Microbial production of renewable fuels or chemicals can be increased by optimization of transport systems (Onyeabor et al. 2020). For example, overexpression of glycerol uptake system Gup1 enhanced glycerol utilization for ethanol in *S. cerevisiae* (Yu et al. 2010). Different efflux pumps were expressed to increase the tolerance of *E. coli* to biofuels and enhance biofuel production (Dunlop et al. 2011).

The FNT family might be utilized in the transportation of organic acids through the plasma membrane of the cells (Soares-Silva et al. 2020). FocA from *E. coli* is for example experimentally identified FNT as a microbial organic acid transporter protein which is known to export acetate, lactate, and pyruvate, uptake/export formate (Wang et al. 2009; Lü et al. 2012). PfFNT from *Plasmodium falciparum* is also another FNT family symporter for Lactate: H^+^ (Wu et al. 2015; Marchetti et al. 2015). Probably the list will be expanded and the manipulation of FNTs for increasing organic acid production in real scales can be seen in the near future.

Another possible use of FNTs seems to be in bio-energy production. Seeking more sustainable methods led to the emergence of hydrogen energy from organic waste materials by using microorganisms. It was shown that cheap glycerol-containing waste can be used as the carbon source to produce H_2_*by E. coli* at a large pH range. Biological energy production such as H_2_, FocA, and FocB functions were studied. It was shown that by designing a membrane by manipulating the original gate control, they force the formate not to exit the cell so that it will be forced to produce H_2_ to be able to reduce its formate concentration inside the cell (Trchounian and Trchounian 2014). The absence of both formate channels may lead to enhanced H_2_ production. Therefore, the growth of *E. coli* on glycerol with the subsequent addition of formate to produce H_2_ is an effective means of producing bio-hydrogen.

### Improving sensor technologies with MTPs

The substrate specificity of porins is inspiring for the invention of novel biosensors. Developing enzyme-based electrode surfaces specifically tailored to transport a single type of ion will help the biosensor to be operated in real samples by largely blocking the entrance of other ions which can cause interference. Electrode stability will also be improved since enzyme inhibition from existing possible inhibitors may also be reduced in this way. Channels can also be preferred for easy transfer of substrates for the development of liposome-based biosensors. Outer membrane protein of *E. coli* (OmpF) was used for such aim for the development of pesticide biosensors in which enzyme acetylcholinesterase was entrapped in liposomes harboring porins allowing entry of organophosphate into the liposome (Yan et al. 2013). NirC coding for nitrite channels of *E. coli* will be used for the development of nitrite-specific biosensors in the near future (Yılmaz et al. 2023). Although there are many known transporters, their mechanism and energetics have not been revealed yet. More research is needed to understand thoroughly the ion transport mechanism of those channels and carriers in different environments in order to improve biosensors based on MTPs.

Promising research on the use of MTPs for biosensor development was performed for the recognition of protein biomarkers in a mixed environment. *Mycobacterium smegmatis* porin A (MspA) nanopore was used to form an electroosmotic flow (EOF) trap which can distinguish different proteins concerning their differently charges such as lysozyme and apo/holo-myoglobin. Besides the trap, an automated event classification was made by extracting multiple event features, and a machine-learning model with a 99.9% accuracy was built by Liu et al. (2022b).

Bacterial aquaporins have been widely used especially in water treatment technologies however there are also challenging developments in biotechnological applications of aquaporins from other organisms. The studies on the use of human aquaporin, Aquaporin 1 (AQP1), for Magnetic Resonance Imaging (MRI), have opened new doors for the use of aquaporins in different areas. Diffusion-weighed MRI encompasses the motility of water molecules by tissues with different structural moieties. In recent years, AQP1 has been employed as a reporter gene for diffusion-weighed MRI to monitor live gene expression in optically opaque animals. In contrast to the reliance of metal-binding proteins on the presence of metal ions and the relatively low resolution of chemical exchange probes, AQP1 showed promising results without hampering the viability of the cells (Mukherjee et al. 2016).

## Biomimetic desalination membranes

Distillation has been the conventional method for seawater desalination since prehistoric times (Angelakis et al. 2021). Being an extremely energy intense and fouling-prone process, distillation has been largely replaced by membrane filtration technology (Van der Bruggen and Vandecasteele 2002). Despite up to 2 to 3 times lower energy requirements compared to distillation (Toth 2020), utilization of energy-efficient pumps, and improved membrane design, reverse osmosis still has a high energy requirement compared to the theoretical minimum accounting for nearly 60% of all operational costs (Elimelech and Phillip 2011; Kumar et al. 2011; Kaufman et al. 2011).

Extremely high water transport rates of aquaporins while rejecting most solutes attracted a great deal of attention from desalination membrane communities within the last decade. Biomimetic membranes, incorporating aquaporins (ABM) have been extensively studied for forward and reverse osmosis filtration systems (Wang et al. 2011a; Zhao et al. 2012). Aquaporins of bacterial origin, such as AqpZ from *E. coli*, can be produced at relatively high expression levels to be incorporated in lipid (Li et al. 2012) or lipid-like biomimetic block copolymers (Kumar et al. 2007) for reduction of energy requirements, enhancing water transport rates and solute rejection. While *E. coli* AqpZ is by far the most commonly used aquaporin in desalination membranes, other microbial aquaporins such as *H. elongata* Aqp (Çalıcıoğlu et al. 2018) and *Photobacterium profundum SS9* Aqp (Wei et al. 2017) have been evaluated for desalination membrane systems yielding a similar water filtration and solute rejection performance.

Interfacial polymerization is the most commonly used technique for the fabrication of ABM, in which aquaporins are incorporated in liposomes or block copolymers and mixed with m-phenylene-diamine in an aqueous solution (Zhao et al. 2012). While water permeability was increased by over 50%, solute rejection remained 17% less of the control membrane void of aquaporin (Qi et al. 2016). Patents granted (Chuyang et al. 2014) and commercialization for ABM indicate the utility of this technology for reverse osmosis (RO) membranes. A commercial FO membrane based on aquaporin is produced by Aquaporin A/S, Lynby, Denmark (Xia et al. 2017) and widely adopted in ABM research (Nikbakht Fini et al. 2020; Zhao et al. 2022b; Chen et al. 2023). ABMs further strengthen the permeate flux, solute selectivity, and anti-fouling capability of FO membranes (Grzelakowski et al. 2015; Camilleri-Rumbau et al. 2019; Chen et al. 2023). Studies on the modification of membranes with biological molecules have increased during the last two decades and new usage areas have been discovered including desalination, dewatering, greywater treatment, or micropollutant removal (Tang et al. 2013; Valverde-Pérez et al. 2020; Yılmaz and Özkan 2022; Chen et al. 2023b). In recent years, coupling FO-ABMs with membrane bioreactors for activated sludge process for wastewater treatment (Luo et al. 2018) or microbial fuel cells (Zhao et al. 2022a, b) for energy generation gained popularity due to excellent pollutant rejection performance and the low fouling propensity of ABMs.

### Use of computational applications for exploring MTPs

Development of computational resources and efficient algorithms, and the availability of online large genome databases have resulted in the enhancement of molecular dynamics simulations such as predicting the structure and function of proteins, docking of channel/transporter-ligand interaction, and immersion of docked complex into a membrane environment (Shiref et al. 2021). Predicting functions of transport proteins with the deep learning approach detecting transport protein genes in large-scale genomes is very useful for the discovery of novel transport proteins (Wang et al. 2023). Over 200 prokaryotic microorganisms’ genome was analyzed for transport proteins by Ren and Paulsen (2007). Up to a thousand membrane transport proteins can be found in the genome of a prokaryotic microorganism, which corresponds to 10 to 13% of the genome.

Computational analysis makes it possible to investigate the effect of environmental conditions on microbial transport protein genes. Microbial metagenomic and meta-transcriptomic data analysis for the high number of prokaryotes in the marine environment shows that the presence of specific transporter traits guides the succession of these microorganisms (Hagström et al. 2021). Generally, environmental organisms such as *Bacillus* spp. and *Pseudomonas* spp. were shown to present the highest number of sodium-dependent pumps as compared to the organisms with autotrophic lifestyles (Ren and Paulsen 2007).

Mapping and gene alignment software tools are widely used for noticing minor differences among gene sequences. Studies show that subtle amino acid substitutions can modulate critical properties of mass transport of the two highly homologous porins. The differences in the protein composition of OmpF/OmpC of *E. coli* were mapped to the respective environmental conditions they are expressed. Results from molecular simulations align well with experimental single-channel measurements (Milenkovic et al. 2023). In addition, knowledge from structural and functional analysis of membrane transport proteins makes *de novo* design of new ion channels possible (Zhou and Lu 2022). By changing amino acid sequences or by combining functional helical barrels, new channels with novel functions can be created (Scott et al. 2021).

### Designing synthetic nanopores mimicking MTPs

Biosensing, drug targeting, and energy production are some of the areas where artificially produced controlled gates with increased efficiency, selectivity, accuracy, etc. are needed. However, it should be noted that the production of such systems is not an easy task due to the fact that proteins are not very robust structures, the channels are narrow for cargo and typically open in response to certain stimuli. An understanding of the mechanism and energetic of natural channels will lead to the inspired designing of synthetic channels which will be game-changers in many fields. Recently, for example, Dey et al. (2022) designed a large and gated channel made via DNA nanotechnology design principles and features. This synthetic channel is expected to allow precisely timed, stimulus-controlled transport of functional proteins across bilayer membranes, which may be used in highly sensitive biosensing, drug delivery of proteins, and the creation of artificial cell networks (Dey et al. 2022).

One of the major limitations of aquaporin desalination membranes is their lability to harsh manufacturing conditions such as high temperatures and the presence of organic solvents (Shen et al. 2015). Therefore, materials mimicking the selectivity and water transport capability of aquaporins are in high demand. Aquaporin-inspired materials include artificial water channels (AWCs), which can be incorporated in lipid or block copolymers similarly to aquaporins while allowing a wider selection of design possibilities. The energetic favourability of artificial water channels co-assembled with peptoid oligomers investigated by using MD simulations suggests that it is possible to improve and use these artificial membranes for desalination purposes (Zhang et al. 2023). Dutta et al. (2023) designed and synthesized artificial oligourea foldamers harboring helical structures that are water-selective. The results are promising that these structures are resistant to proteases and microorganisms and can be used as artificial water channels for water purification purposes (Dutta et al. 2023). Song et al. (2020) designed a cluster-forming organic nanoarchitecture, peptide-appended hybrid [4] arene (PAH[4]), this architecture was shown to enable a highly efficient and selective water permeation through mechanisms distinct from traditional water channels.

Carbon nanofibers have long been suggested as an alternative to aquaporin biomimetic membranes for desalination (Jirage et al. 1997). Recently, Güvensoy-Morkoyun et al. (2022), demonstrated the modification of carbon nanotubes with arginine residues as in the selectivity filter of aquaporins achieving salt rejection and satisfactory water transport rates. A similar approach was used towards a ceramic material, anodic aluminium oxide (Jeon et al. 2023), to obtain a shelf-stable ABM.

Besides, inspiring selective biosensor and biomimetic material design or being a target for a novel drug, nanopores are regarded to have the potential for characterizing proteins and nucleic acids and be used for sequencing of DNA and proteins (Jeong et al. 2022; Milenkovic et al. 2023). The design of membranes harboring specific nanopores can even be used for energy generation. It is suggested that the huge osmotic pressure generated by the salinity gradient at the interface between fresh and salt waters can be converted into blue energy with nanopore-based filtration membranes (Siria et al. 2017).

### Future perspectives

Membrane transport proteins, most of which are specialized for the transport of specific substances, are a large family containing a wide variety of channels and carrier proteins embedded in cellular membranes. New members or new functions of MTPs are being discovered every day, and these discoveries make remarkable contributions to the knowledge about cell machines and the development of technologies that will be inspired by them. Commercialization of the biomimetic desalination membranes, one of the earliest and the most important technologies inspired by microbial channel proteins, increases the hope of the applicability of MTPs for developing novel technologies. This review lists the biotechnological applications of microbial MTPs including designing drugs for disease treatment, improving sensor technologies, increasing the production of fermentation products, etc. Although studying microbial MTPs is easier as compared to the other counterparts in higher-level organisms, extraction of these proteins from membranes still requires costly processes. Therefore, the most exciting future challenge may be the synthesis of artificial molecules mimicking the function of MTPs.

The technological advances provide tools for exploring huge amounts of sequence data and identification of specific sequences that fold into unique 3D protein structures. With the help of computer-assisted 3D simulations and wet-lab studies, *de novo* design of novel channels or pores is now possible. It is not hard to see the future application of specialized channels or transporters with high specificity and selectivity, which will increase the effectiveness of biotechnological processes. In addition, the treatment of infectious diseases by drugs targeting microbial MTPs seems to be ready for acceleration. Developments in the design and production of novel drugs targeting channels and pores are parallel to the advances in technologies for studying the structure and functions of MTPs. The boost in knowledge accumulation due to the development of tools and techniques overcoming the challenges of studying membrane proteins will ease the revealing of the nature and mechanism of MTPs, which will result in a much greater increase in the quantity and variety of future applications.

## Data Availability

No datasets were generated or analysed during the current study.
